# The effects of acute stress on the calibration of persistence

**DOI:** 10.1016/j.ynstr.2017.11.001

**Published:** 2017-11-22

**Authors:** Karolina M. Lempert, Joseph T. McGuire, Danielle B. Hazeltine, Elizabeth A. Phelps, Joseph W. Kable

**Affiliations:** a Department of Psychology, New York University, New York, NY, USA; b Department of Psychological & Brain Sciences, Boston University, Boston, MA, USA; c Nathan Kline Institute for Psychiatric Research, Orangeburg, NY, USA; d Department of Psychology, University of Pennsylvania, Philadelphia, PA, USA

**Keywords:** Acute stress, Persistence, Delay of gratification, Cortisol, Inverted U, Impulsivity

## Abstract

People frequently fail to wait for delayed rewards after choosing them. These preference reversals are sometimes thought to reflect self-control failure. Other times, however, continuing to wait for a delayed reward may be counterproductive (e.g., when reward timing uncertainty is high). Research has demonstrated that people can calibrate how long to wait for rewards in a given environment. Thus, the role of self-control might be to integrate information about the environment to flexibly adapt behavior, not merely to promote waiting. Here we tested effects of acute stress, which has been shown to tax control processes, on persistence, and the calibration of persistence, in young adult human participants. Half the participants (n = 60) performed a task in which persistence was optimal, and the other half (n = 60) performed a task in which it was optimal to quit waiting for reward soon after each trial began. Each participant completed the task either after cold pressor stress or no stress. Stress did not influence persistence or optimal calibration of persistence. Nevertheless, an exploratory analysis revealed an “inverted-U” relationship between cortisol increase and performance in the stress groups, suggesting that choosing the adaptive waiting policy may be facilitated with some stress and impaired with severe stress.

## 1 Introduction

The ability to persist in waiting for future rewards is central to self-control. Yet people often fail to persist in waiting, even when they express a desire for the future reward. For example, many people do not stick to healthy diets even when they have a goal to lose weight. Contextual factors, such as the person's beliefs about the environment, can influence whether an individual persists in waiting for future rewards. For instance, if a person believes that not having seen results in a week means they are unlikely to lose weight at all, they may give up on their diet. Another potentially relevant contextual factor is one's ongoing level of stress. It is unknown how stress affects overall levels of persistence or how it interacts with beliefs about the environment. The present study tests how acute aversive stress, induced by the cold pressor test, affects subsequent decisions about waiting for future rewards.

Stress can be defined in multiple ways, but here we focus on a relatively long-lasting affective state that is characterized by specific physiological and neurohormonal changes. A stress reaction is accompanied by transient sympathetic nervous system arousal, as well as activation of the hypothalamic-pituitary-adrenal (HPA) axis, which results in the release of glucocorticoids, such as cortisol (Arnsten, 2009, Joëls and Baram, 2009). These neurohormonal effects of stress, which can persist for minutes to hours following the stressor (Dickerson and Kemeny, 2004), have been shown to impair cognitive capacities that depend on the prefrontal cortex (PFC; Arnsten, 2009, Holmes and Wellman, 2009), including goal-directed behavior (Otto et al., 2013, Plessow et al., 2012) and executive control and flexibility (Alexander et al., 2007, Goldfarb et al., 2016, Plessow et al., 2011). Here we measure cortisol as a marker of HPA-axis activation following stress, and investigate whether stress influences subsequent persistence for delayed rewards.

The precise role of PFC-mediated cognitive control (and consequently, the precise effect of stress) in persistence decisions is subject to debate. One perspective holds that the ability to keep waiting through a delay depends on sustaining self-control, or “willpower,” amid a dynamic interplay between “hot” and “cool,” or “affective” and “deliberative” mental processes (Metcalfe and Mischel, 1999). In this framework, successful persistence relies on exerting cognitive control (activating the “cool” system) in order to combat temptation (which increases activity in the “hot” system). There is some evidence for this perspective; in the famous “marshmallow experiment,” children who were able to distract themselves from the food items in front of them in order to reduce their emotional impact were more successful at waiting for the experimenter to return (Mischel et al., 1972). Moreover, the prefrontal cortex has been shown to be involved in exerting control to avoid temptations in some contexts (Hare et al., 2009, Maier et al., 2015). This “hot”/“cool” theory predicts that acute stress would lead to a reduced tendency to wait for future rewards, by taxing PFC-dependent cognitive control and shifting the balance of activity toward a “hot” motivational system (Heatherton and Wagner, 2011, McClure and Bickel, 2014).

An alternative theory for how persistence decisions are approached makes a different prediction about how acute stress would influence this process. It has recently been proposed that the decision to persist emerges from a dynamic reassessment of costs and benefits that takes into account one's beliefs about the environment (McGuire and Kable, 2013). In other words, people continually re-evaluate the subjective value of an awaited reward based on how long they have been waiting and how long they believe they still have to wait. In certain situations (including many that require self-control), when uncertainty about future reward timing is high, it may be adaptive to quit waiting after a period of time. In a set of studies, McGuire and Kable, 2012, McGuire and Kable, 2015 showed that people are able to calibrate their waiting times based on the statistics of the reward environment. Specifically, people wait longer when reward delays are drawn from a uniform distribution and are sure to arrive within a predictable period of time (“high-persistence” environment), and they wait less time when reward delays are drawn from a heavy-tailed distribution, when it is suboptimal to wait for every delayed reward (“limited-persistence” environment). The ventromedial prefrontal cortex (vmPFC) has been linked with the dynamic valuation signal that enables calibrating waiting times appropriately (McGuire and Kable, 2015). Thus, according to this dynamic reassessment hypothesis, the role of PFC-dependent cognitive control is not to increase persistence, but rather, to flexibly calibrate persistence behavior according to one's knowledge about the timing statistics of the environment. If acute stress impaired this calibration process, the result would be reduced waiting time in “high-persistence” conditions, but *increased* waiting time in “limited-persistence” conditions.

A third possible outcome is that acute stress would have no overall effect on persistence or the calibration of persistence. Indeed, one study showed that acute sleep deprivation (another type of psychophysiological perturbation) did not significantly influence persistence decisions (Massar and Chee, 2015). Often null findings of stress may emerge because of individual differences in stress-response magnitude combined with an underlying non-monotonic dose-response function. Behavior in tasks that rely on the PFC has been shown to suffer under high levels of stress but *improve* under low levels of stress (Diamond et al., 2007, Luksys and Sandi, 2011, Sapolsky, 2015). For example, model-based learning, which involves bearing a complex task structure in mind, is impaired under stress but only in individuals with low working-memory capacity, for whom the task is more difficult (Otto et al., 2013). This “inverted-U” pattern, if found here, would mask any overall effect of stress on behavior. Given the preponderance of evidence that performance in goal-directed tasks after acute stress follows an “inverted-U” function, in the present work we tested both linear and curvilinear models to relate individual stress responses to behavior.

In the current study, we tested three possible effects of stress on persistence behavior. We induced stress with the cold pressor test, a manipulation that involves submerging an individual's arm in ice water for 3 min. If stress interferes with control processes necessary for persistence in the face of a delay, then acute stress should impair the ability to wait for delayed rewards. If instead stress interferes with control processes that support the optimal calibration of waiting time depending on the statistics of the environment, then high levels of acute stress would interfere with the ability to wait in high-persistence conditions, but would lead to excessive waiting in limited-persistence conditions. Finally, it may be that both overall persistence and the calibration of persistence are impervious to the effects of acute stress.

## 2 Methods

### 2.1 Participants

One hundred and twenty participants (69 F; mean age = 23.34; SD = 4.04; 30 participants per group, consistent with previous studies of stress and decision-making: FeldmanHall et al., 2015, Lenow et al., 2017, Otto et al., 2013) were recruited via paid advertisement on New York University's campus and received $15/hour for participating in the study, in addition to compensation from the task (∼$10; see below for details). Approval was obtained from the University Committee on Activities Involving Human Subjects at New York University, and all participants signed a consent form before the experiment.

### 2.2 Procedure

To control for circadian fluctuations in cortisol levels (Lupien et al., 2007), all sessions were conducted between the hours of 12 and 5 p.m. Subjects were randomly assigned to one of four groups, representing a 2 × 2 crossing of a stress manipulation (stress vs. no stress) and a manipulation of the timing in the willingness-to-wait task: Stress High Persistence, Control High Persistence, Stress Limited Persistence and Control Limited Persistence.

After giving informed consent, participants completed a pre-study questionnaire, which assessed factors that might influence the stress response, including current medication use (corticosteroids, beta-blockers, anti-depressants, and oral contraceptives) and routine exposure to ice baths. After 7 min of acclimation to the lab environment, subjects provided the first saliva sample (T1) and then completed three questionnaires: the Perceived Stress Scale (PSS; Cohen et al., 1983) which measures the extent to which stressors have felt uncontrollable in the last month, the Beck Depression Inventory (BDI-II; Beck et al., 1996), which measures depressive symptoms, and the State and Trait Anxiety Inventory - Trait version (STAI-T; Spielberger, 1983), which measures the participant's general susceptibility to be anxious.

**Fig. 1:**
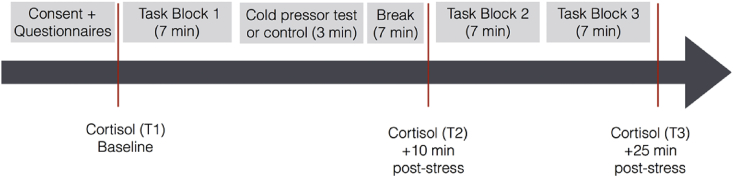
Timeline of the procedure. After an acclimation period during which participants gave informed consent, the first cortisol measurement was taken. Then subjects did one block of the willingness-to-wait task before they underwent either the cold pressor test (stress groups) or submerged their arms in room temperature water (control groups). After a short break during which cortisol levels increase after acute stress, another cortisol measurement was taken. Following this, participants completed two more blocks of the willingness-to-wait task before the final cortisol measurement.

Upon their completion of the questionnaires, participants were presented with Block 1 (the first of three) of the willingness-to-wait task (described below). This first block was completed prior to the stress manipulation, to allow learning of the task to stabilize prior to the stress or control manipulation. Stress has been found to influence learning processes (Luksys and Sandi, 2011), and here we were interested in stress effects on performance, not initial learning. Participants then completed the Positive and Negative Affect Scale (PANAS; Watson et al., 1988) to assess current levels of positive and negative affect. Subjects then underwent either the stress or control manipulation (described below). Following this, participants completed a second PANAS questionnaire to assess how their affect changed after experiencing the stress or control manipulation. After this, there was a 7 min break to allow for cortisol levels to increase in the stress groups (Dickerson and Kemeny, 2004). The second saliva sample (T2) was taken at the end of this break period, before Blocks 2 and 3 of the task were presented to the participant. The third and final saliva sample (T3) was taken after the task was completed. Finally, participants completed three more questionnaires: the Deferment of Gratification scale (DoG; Ray and Najman, 1986), which measures ability to wait for rewards in everyday life, the Barratt Impulsiveness Scale (BIS; Patton et al., 1995) which measures everyday impulsiveness, and the Intolerance of Uncertainty questionnaire (IUS; Buhr and Dugas, 2002) which measures attitudes toward uncertainty in everyday life. Fig. 1 depicts the timeline of the procedure.

### 2.3 Stress manipulation

Participants in the stress groups underwent the cold pressor test (CPT), during which they submerged their non-dominant hand to elbow in a 0–4 °C ice-water bath for 3 min. The CPT is widely used in the laboratory to model the effects of mild to moderate acute stress that participants might encounter in everyday life. It has been shown to reliably activate sympathetic nervous system and hypothalamic-pituitary-adrenal axis arousal as measured by increased physiological, endocrine (i.e., cortisol), and subjective levels of stress (Lupien et al., 2007, McRae et al., 2006, Velasco et al., 1997). Participants in the control groups submerged their non-dominant arms in room temperature water for 3 min. To assess subjective levels of stress after the task, all participants rated how unpleasant they found the stress or control task on a scale from 0 (not at all unpleasant) to 10 (extremely unpleasant).

### 2.4 Cortisol measurement

We measured cortisol levels from saliva samples. As salivary free cortisol levels correspond well with plasma free cortisol levels, collection of saliva is a simple, non-invasive means to obtain an index of the biologically active fraction of this hormone (Kirschbaum and Hellhammer, 1989). Participants were instructed to refrain from eating, consuming caffeine and alcohol, and exercising for at least 2 h before study participation. Saliva samples were collected using Salimetrics oral swabs after the first set of questionnaires and acclimation to the lab environment (T1), and approximately 10 min (T2) and 25 min after the stress manipulation (T3). Cortisol levels corresponding to the stress response tend to peak at around 20–30 min post-stress (Dickerson and Kemeny, 2004, Kirschbaum et al., 1993). Subjects placed the oral swabs under their tongues for 2 min, after which the swabs were placed in vials and stored in a freezer until later processing. Cortisol samples were assayed at Salimetrics, LLC (Carlsbad, CA, USA). A participant was considered an outlier if his/her raw baseline cortisol measurement was more than 2.3 standard deviations from the mean; outliers were excluded from all analyses.

To assess individual differences in cortisol change in response to stress, we first log_10_-transformed the raw cortisol measurements (which were in units of μg/dl). This is common practice in stress research (Lenow et al., 2017, Otto et al., 2013), because cortisol measurements tend to be non-normally distributed (Miller et al., 2013). Then we subtracted the cortisol measurement at T1 from the average of cortisol measurements at T2 and T3. This “Δ Cortisol” measure represents the amount of cortisol secreted as a result of the manipulation (Lenow et al., 2017, Otto et al., 2013).

### 2.5 Willingness-to-wait task

This task has been previously used to study persistence (McGuire and Kable, 2015, McGuire and Kable, 2012) and was programmed using the Psychophysics Toolbox in Matlab (Brainard, 1997, Kleiner et al., 2007). At the outset of the task, participants were told that their goal was to earn as much money as possible in a fixed amount of time (7 min per block), since they would keep any money that they earned in the task as additional compensation for the study.

**Fig. 2:**
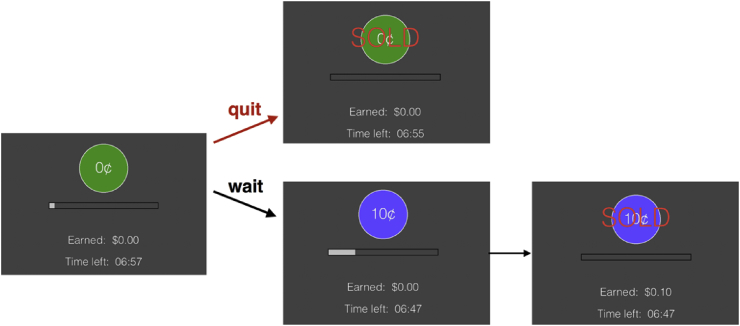
One trial of the willingness-to-wait task. Subjects could either wait for the token to mature to sell it, or sell the token before it matured to advance to the next trial. Once they pressed a key to sell the token, the word “SOLD” appeared over the token for 1 s, and then, after a blank 1-s ITI, a new trial appeared on the screen. A visual representation of time was depicted by a moving bar below the token. Time left (out of 7 min) was also indicated on each screen.

On each trial of this task, participants saw a circular green token labeled “0¢” appear on the screen. After a random delay, the token turned blue and was worth 10¢. Participants could sell the token at any time by pressing the spacebar. After pressing the spacebar, they were shown feedback (the word “SOLD” appeared over the token) for 1 s. After a 1 s blank ITI, a new trial began (see Fig. 2 for sample trial). The token's value was added to the participant's total earnings, which accumulated throughout the block. The earnings were displayed on the screen throughout the experiment, along with the time remaining in the block. A white progress bar marked the amount of time the current token had been on the screen, and the full length of the bar corresponded to 50 s. The bar grew continually from the left and re-set when a new token appeared. The progress bar was included to discourage a strategy of counting time (McGuire and Kable, 2015). Participants were informed that they could sell the token before it matured if they felt it was taking too long and they wanted to move on to a new token, but they would not earn any money from selling 0¢ tokens. Unlike in a previous version of this task (McGuire and Kable, 2012), the initial token was not worth any money. This has little influence on behavior or the incentive structure of the task (McGuire and Kable, 2015), and it ensures that participants do not spend time exploring the ultimately unproductive strategy of ignoring the large reward and solely accruing small rewards.

**Fig. 3:**
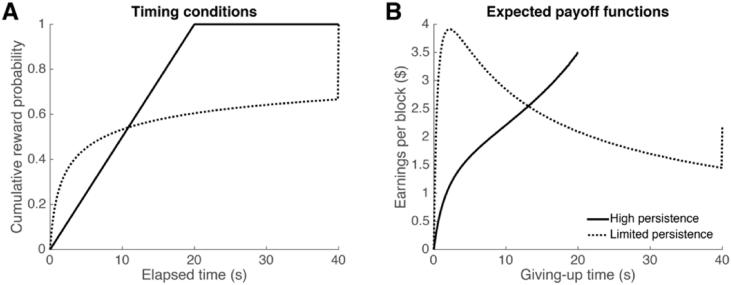
(A) Cumulative reward probability as a function of elapsed time in the trial in the high persistence and limited-persistence conditions. In the high persistence condition, all delays were between 0 and 20 s, and the cumulative probability of reward receipt increased linearly until 20 s had passed. In the limited-persistence condition, the cumulative reward probability increased steeply in the first few seconds, and then less dramatically from there on out. (B) Projected (average) earnings per block as a function of giving-up time in each trial. The reward rate increased in the high persistence condition as the giving-up time increased, suggesting that it is optimal to never give up until the reward arrives. In the limited-persistence condition, on the other hand, the average reward rate increased until approximately 2.22 s, and then began to decrease, so the optimal strategy was to quit if the reward had not arrived in that amount of time.

Each participant was randomly assigned to either a high-persistence or limited-persistence task condition. These conditions differed in the timing statistics of reward delivery, and were designed so that either high or limited persistence was advantageous. In the high-persistence condition, delays were drawn from a uniform distribution, spanning 0–20 s. Here the reward maximizing strategy was to always wait for the token to mature. In the limited-persistence condition, delays were drawn from a (truncated) generalized Pareto distribution. In this heavy-tailed distribution, there was a high probability that the token would mature after a short delay, but after that delay had passed, the token was unlikely to mature until 40 s had passed. Here the optimal waiting policy was to quit if the token had not arrived within 2.22 s (see *2.6. Normative Analysis* section below). After 2.22 s, the subjective value of waiting deteriorated since the opportunity cost of waiting for the token to mature increased (Fig. 3).

### 2.6 Normative analysis

The “giving-up time” is defined as the time at which a decision maker will give up waiting on each trial if the reward has not yet arrived. The expected return for giving up at time *t* is calculated as follows. Let *p*_t_ be the proportion of rewards delivered earlier than *t*. Let τ_t_ be the mean duration of these rewarded trials. One trial's expected return, in dollars per second, is:

$$R_{t}\;=\frac{0.10\left(p_{t}\right)\;}{{\text{τ}}_{t}\;p_{t}+\text{ t }\left(1\;-\;p_{t}\right)\;+\;2\;}$$

The numerator is the trial's expected gain in dollars, and the denominator is the trial's expected cost in seconds, given a 10¢ reward and 2-s ITI. The value of *t* that maximizes R_t_ is the optimal giving-up time. The optimal giving-up time in the high-persistence condition was 20 s (reward rate = 83¢ s^−1^), while the optimal giving-up time in the limited-persistence condition was 2.22 s (reward rate = 93¢ s^−1^).

### 2.7 Behavioral measures

To operationalize participants' willingness to wait, we constructed a Kaplan–Meier survival curve for each participant. The Kaplan–Meier is a nonparametric estimator of the survival function (Kaplan and Meier, 1958). For each time *t*, it plots the participant's probability of waiting at least until *t* if the reward is not delivered earlier. Analyses were restricted to the 0–20 s interval for which there were observations in both conditions (we can only observe an individual's willingness to wait *t* seconds if we have trials where the scheduled delay equals or exceeds *t*). The area under the survival curve (AUC) is a useful summary statistic, representing the average number of seconds an individual was willing to wait within the analyzed interval. We constructed two Kaplan-Meier curves and computed the AUC separately for each. The first curve corresponded to behavior in the first block of the task (pre-manipulation) and the second corresponded to behavior in the last two blocks of the task (post-manipulation).

To assess the calibration of persistence, we calculated the deviation from the optimal giving-up time, the extent to which participants waited too long in the limited-persistence condition or not long enough in the high-persistence condition. In the high-persistence group this corresponded to 20 s minus the participant's AUC; in the limited-persistence group it corresponded to the participant's AUC minus 2.22 s. In the latter group, we excluded the 5 participants (out of 60) whose AUC was lower than 2.22. Thus, a large deviation from optimal is indicative of waiting too long in the limited-persistence condition, and not waiting long enough in the high-persistence condition. As a secondary measure of performance in the blocks post-manipulation, we also recorded the total earnings (in dollars) in those blocks, expecting that better-calibrated individuals would tend to earn more money.

## 3 Results

### 3.1 Pre-study exclusionary criteria questionnaire

In the pre-study questionnaire, 2 participants reported routine exposure to ice baths (as part of athletic training), but since they were in the control (i.e., warm water) group, they remained in the sample. No participants reported current corticosteroid, beta-blocker, or anti-depressant use. Sixteen participants reported current oral contraceptive use (10 in stress group, 6 in control group). Whereas they were not excluded, all analyses of cortisol data were also done without these participants, since there is some evidence that cortisol responses to stress may be influenced by oral contraceptive use (Kuhlmann and Wolf, 2005, Rohleder et al., 2003).

One participant (in the stress group) had a baseline cortisol measurement more than 5 SD from the mean (1.132 μg/dl) so they were removed from all analyses, leaving 119 participants.

### 3.2 Manipulation check

The stress manipulation had a significant effect on subjective markers of stress (see Table 1). We used the PANAS scale to assess subjective affect. In a 2 × 2 ANOVA with stress group (Stress/No Stress) as a between-subjects factor and time (pre/post manipulation) as a within-subjects factor, negative affect showed borderline main effects of time (F_(1,117)_ = 4.03; *p* = 0.047) and group (F_(1,117)_ = 4.06; *p* = 0.046). Negative affect tended to increase over time, and the stress group endorsed higher negative affect ratings overall. Crucially, however, there was a significant group × time interaction (F_(1,117)_ = 13.33; *p* < 0.001). Independent paired *t*-tests revealed no significant difference in negative affect between groups before the stress or control manipulation (t_117_ = 0.08; *p* = 0.936), but a significant difference after the manipulation (t_117_ = −3.40; *p* = 0.001). There was a main effect of time on positive affect (F_(1,117)_ = 7.13; *p* = 0.009), such that positive affect decreased after the manipulation. There was only a trending main effect of group (F_(1,117)_ = 3.43; *p* = 0.067), and interaction of group and time (F_(1,117)_ = 3.65; *p* = 0.059) on positive affect ratings, consistent with previous research showing that stress does not necessarily lower positive affect ratings, even as it increases negative affect ratings (Ossewaarde et al., 2011).

**Table 1:** Affective ratings and raw cortisol values for control and stress groups.

Measure	Control Group (N = 60)	Stress Group (N = 59)	Control > Stress
Positive Affect (pre-manipulation)	M = 32.72 SD = 7.52	M = 29.98 SD = 6.71	t_117_ = 2.78 p = 0.006
Positive Affect (post-manipulation)	M = 30.17 SD = 9.74	M = 28.66 SD = 8.32	t_117_ = 0.91 p = 0.367
Negative Affect (pre-manipulation)	M = 14.50 SD = 3.86	M = 14.42 SD = 6.18	t_117_ = 0.08 p = 0.936
Negative Affect (post-manipulation)	M = 13.68 SD = 4.34	M = 17.24 SD = 6.82	t_117_ = −3.40 p < 0.001
Manipulation unpleasantness rating	M = 1.52 SD = 2.10	M = 7.76 SD = 2.12	t_117_ = −16.13 p < 0.001
Baseline Cortisol (μg/dl)	M = 0.22 SD = 0.13	M = 0.21 SD = 0.15	t_117_ = 0.77 p = 0.443
Cortisol +10 min post manipulation (μg/dl)	M = 0.19 SD = 0.10	M = 0.25 SD = 0.14	t_117_ = −2.96 p = 0.004
Cortisol +25 min post manipulation (μg/dl)	M = 0.21 SD = 0.17	M = 0.39 SD = 0.28	t_117_ = −5.08 p < 0.001

Mean ratings for positive and negative affect pre- and post-manipulation, mean unpleasantness ratings (scale: 0–10, where 0 = neutral; 10 = extremely unpleasant) for the water bath manipulation, and raw cortisol values at baseline, 10 min after manipulation and 25 min after manipulation. All *t*-tests are two-sided. Note: summary statistics for cortisol are shown in raw units, but significance testing was performed on log-transformed values. Stress groups showed a significant increase in negative affect and cortisol compared to control groups following the stress manipulation, and they endorsed significantly higher unpleasantness ratings.

After the CPT or control manipulation, participants were asked how unpleasant they found the water bath, on a scale from 0 to 10. There was a main effect of stress group on unpleasantness rating (F_(1,117)_ = 260.29; *p* < 0.001), in that individuals who underwent the CPT reported the water bath as significantly more unpleasant compared to individuals who underwent the room temperature water bath in the control condition (t_117_ = −16.13; *p* < 0.001).

**Fig. 4:**
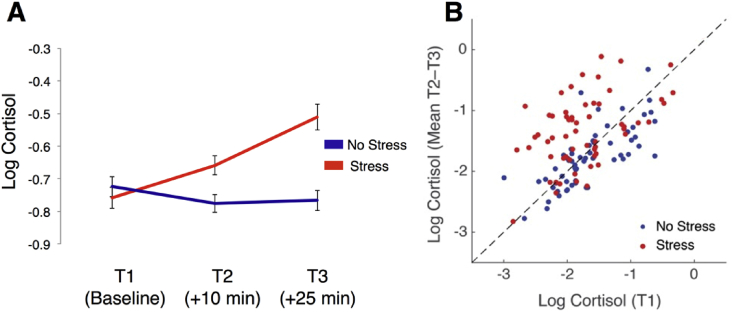
Effects of stress manipulation on cortisol levels. (A) Average (log_10_) cortisol at baseline, at 10 min after the manipulation, and at 25 min after the manipulation for stress and control groups. Cortisol was higher for individuals in the stress groups at T2 and T3, but not at baseline. (B) Scatterplot showing cortisol at baseline plotted against average cortisol across T2 and T3. Individuals to the left of the diagonal showed higher cortisol levels post-manipulation. While there were large individual differences in cortisol responsiveness, individuals in the stress group tended to show increases from baseline.

The stress manipulation also had a significant effect on physiological markers of stress. We examined cortisol measurements as a physiological measure of stress response. We conducted a 2 × 3 ANOVA with stress group (Stress/No Stress) and time (Baseline / 10 min post-manipulation/ 25-min post-manipulation) as factors. There was a significant effect of time (F_(2, 1.37)_ = 14.57; *p* < 0.001) and of group (F_(1,117)_ = 8.47; *p* = 0.004) on cortisol, in that cortisol levels increased over time overall, and were higher in the stress group compared to the control group. Critically, however, there was a significant time × group interaction (F_(2, 1.37)_ = 26.50; *p* < 0.001) on cortisol. Individuals in the stress groups had significantly higher cortisol levels 10 min (t_117_ = −2.96; *p* = 0.004) and 25 min post manipulation (t_117_ = −5.08; *p* < 0.001), but not at baseline (t_117_ = 0.44; *p* = 0.443; Fig. 4). All effects remained significant when excluding the sixteen participants who reported current oral contraceptive use (main effect of group: F_(1,101)_ = 5.86; *p* = 0.017; main effect of time: F_(2, 1.38)_ = 15.13; *p* < 0.001; group × time interaction: F_(2, 1.38)_ = 28.09; *p* < 0.001).

### 3.3 No effects of stress manipulation on persistence or calibration of persistence

We replicated previous research (McGuire and Kable, 2015, McGuire and Kable, 2012) showing that individuals are capable of learning the statistics of the environment and calibrating their waiting behavior appropriately. We conducted a 3-way ANOVA, with group (Stress / No Stress) and task condition (Limited-Persistence / High Persistence) as between-subjects factors, Time (Pre / Post manipulation) as a within-subjects factor, and the AUC of the Kaplan-Meier survival curve as the dependent variable. There was a main effect of task, showing that individuals in the limited-persistence condition successfully waited less time on average than individuals in the high-persistence condition (F_(1, 115)_ = 33.71; *p* < 0.001). There was a main effect of time, indicating that across all participants, people were more likely to wait less time after the manipulation than before (F_(1, 115)_ = 19.30; *p* < 0.001). Finally, there was a time × task interaction, showing that individuals were learning over time to wait less in the limited-persistence condition relative to the high-persistence condition (F_(1, 115)_ = 18.15; *p* < 0.001). After the manipulation, participants in the high-persistence condition waited longer than participants in the limited-persistence condition, both in the stress group (mean difference between high-persistence and limited-persistence = 6.45 s; SE of difference = 1.30 s; t_57_ = 4.95; *p* < 0.001) and the control group (mean difference between high-persistence and limited-persistence = 5.96 s; SE of difference = 1.35 s; t_58_ = 4.40; *p* < 0.001).

**Fig. 5:**
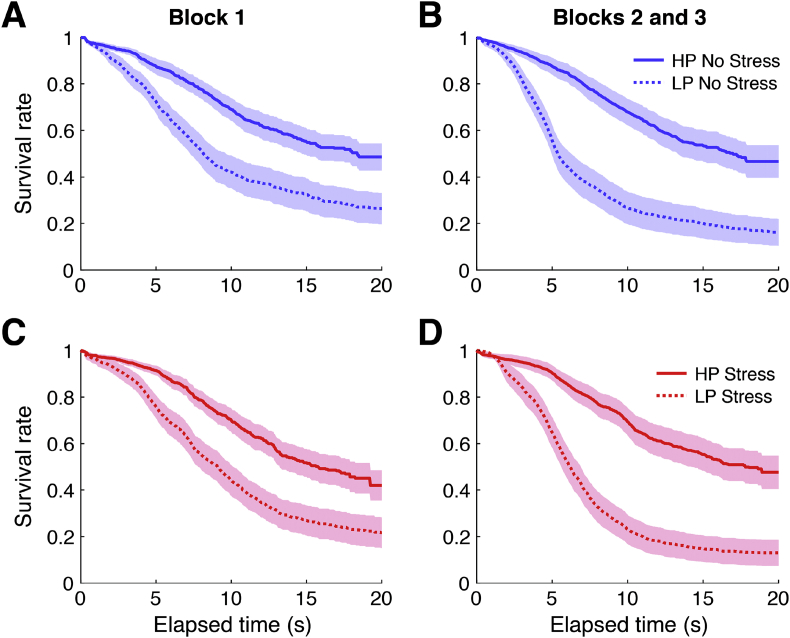
Average Kaplan-Meier survival curves for participants in the control (Panels A, B) and stress groups (C, D). Solid lines represent participants in the high-persistence (HP) condition. Dotted lines represent participants in the limited-persistence (LP) condition. Shaded areas signify SEM. While differences between high-persistence and limited-persistence conditions increased over time with experience in the task (Area under the curve (AUC) of the Kaplan-Meier curve increased in the high-persistence condition and decreased in the limited-persistence condition), there were no overall differences between the stress and control groups in behavior in this task, either with respect to calibration of persistence, or persistence in general.

Contrary to our initial hypotheses, acute stress interfered neither with the ability to wait for future rewards nor with the ability to optimally calibrate persistence based on the task environment. There was no main effect of stress on giving-up time (F_(1, 115)_ = 0.002; *p* = 0.966), nor was there a stress × time interaction (F_(1, 115)_ = 0.05; *p* = 0.821), showing that stress did not make individuals more or less likely to persist for delayed rewards. There was also no stress × task interaction (F_(1, 115)_ = 0.01; *p* = 0.915), or stress x task × time interaction (F_(1, 115)_ = 0.29; *p* = 0.590; Fig. 5), showing that the ability to calibrate persistence appropriately was also intact under stress.

### 3.4 Exploratory: curvilinear relationship between cortisol response to stress and calibration of persistence

Although there was no effect of the stress manipulation on overall persistence or calibration of persistence in the willingness-to-wait task, previous research has shown that the magnitude of the physiological response to stress can predict the extent of behavioral effects. Specifically, there is evidence for both linear (Goldfarb et al., 2016, Luksys et al., 2009, Maier et al., 2015) and curvilinear (Diamond et al., 2007, Luksys and Sandi, 2011, Otto et al., 2013, Sapolsky, 2015) relationships between cortisol responses to stress and behavior in PFC-dependent tasks. Such effects might be masked if only overall effects of the stress manipulation are considered. Thus, in an exploratory analysis, we examined individual differences in behavior as a function of stress response. To this end, we computed Δ Cortisol for each participant, and collapsed across both timing manipulation groups within the stress group to increase power. We excluded participants who did not show an increase in cortisol post-stress (i.e., Δ Cortisol < 0, N = 20; leaving N = 36^2^).

To test for effects of Δ Cortisol on giving-up time, we entered AUC post-manipulation as the dependent variable in our regression. We controlled for timing condition (High-Persistence or Limited-Persistence) by entering it as a dummy variable. To test for effects of Δ Cortisol on the calibration of persistence, we used deviation from optimal giving-up time as our dependent variable. We tested both linear and quadratic models.

Contrary to the hypothesis that acute stress decreases persistence in the face of a delay, there was no discernible effect of the physiological response to stress on people's ability to wait for delayed rewards. There was neither a significant linear relationship between Δ Cortisol and AUC (β = −3.36; *p* = 0.473), nor a significant quadratic relationship (Δ Cortisol β = 3.57; *p* = 0.829; (Δ Cortisol)^2^ β = −11.68; *p* = 0.663).

**Fig. 6:**
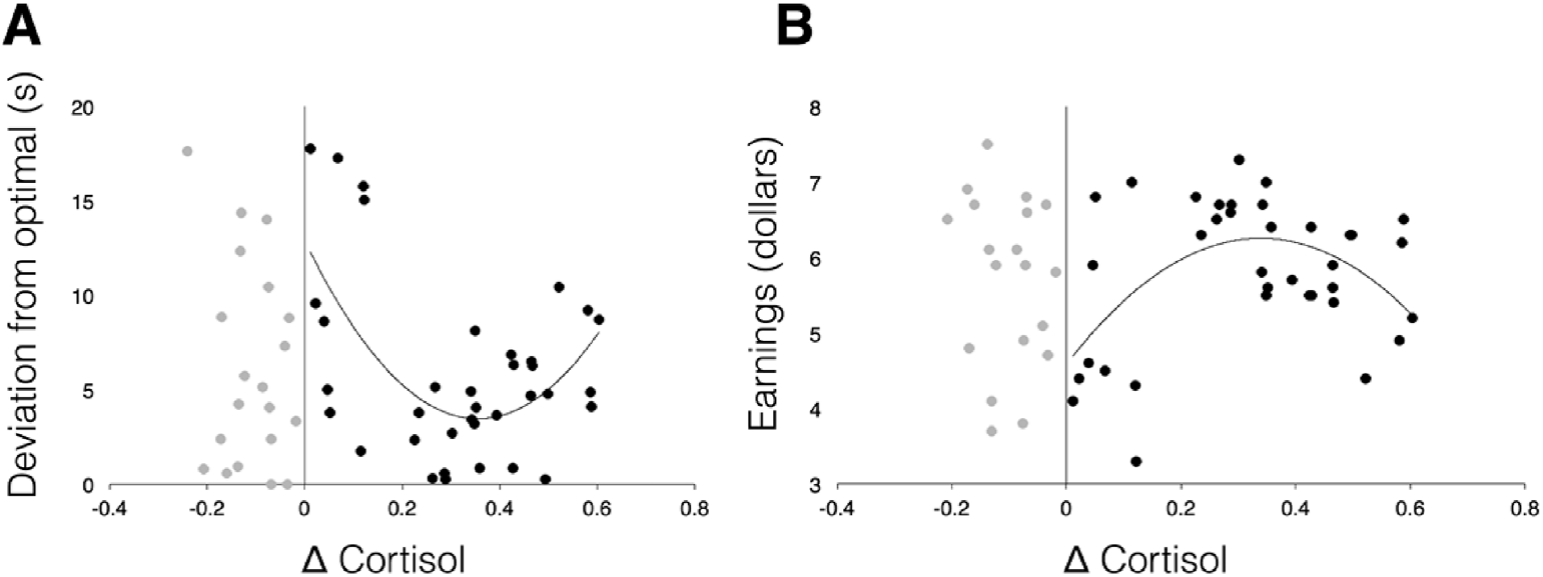
Plots of Δ Cortisol against deviation from optimal giving-up time (A) and earnings (B) in the stress groups. Individuals in gray had cortisol responses to the stressor that did not exceed zero, so they were not included in analyses. Among participants with an increase in cortisol (shown in black), there was a significant curvilinear relationship between these variables and Δ Cortisol (deviation from optimal: F_(2,33)_ = 8.51; *p* = 0.001; earnings: F_(2,33)_ = 5.69; *p* = 0.008).

We did find, however, some support for the hypothesis that acute stress would influence the ability to flexibly calibrate persistence. Specifically, there was an inverted-U shaped relationship between people's physiological response to stress and their ability to calibrate persistence appropriately to the environment. There was a trend suggesting a linear relationship between Δ Cortisol and deviation from optimal giving-up time (β = −8.49; *p* = 0.054; R^2^ = 0.1046; AIC = 212.52). However, the quadratic model fit the data significantly better (Δ Cortisol β = -52.98; *p* < 0.001; 95% CI {-80.41, −25.55}; (Δ Cortisol)^2^ β = 74.57; *p* = 0.002; 95% CI {30.38, 118.77}; F_(2,33)_ = 8.51; *p* = 0.001; R^2^ = 0.3403; AIC = 203.53; likelihood ratio test comparing models: *χ*^2^ = 10.99; *p* < 0.001; Fig. 6a). Among responders to the stress manipulation, when cortisol increased slightly post-stress, individuals performed more optimally in the task – they waited less time for rewards in the limited-persistence condition, and more time for rewards in the high-persistence condition. However, responders with a more substantial increase in cortisol post-stress performed worse. This relationship did not hold when examining the control groups, showing that random fluctuations in cortisol do not yield this same behavioral pattern (Δ Cortisol β = 3.37; *p* = 0.426; (Δ Cortisol)^2^ β = 6.31; *p* = 0.663; F_(2,56)_ = 0.38; *p* = 0.6833; R^2^ = 0.0135; AIC = 365.67). Moreover, both linear and quadratic relationships did not hold when examining non-responders within the stress group (Δ Cortisol < 0; N = 20; linear: Δ Cortisol β = −17.53; *p* = 0.396; quadratic: Δ Cortisol β = 27.66; *p* = 0.735; (Δ Cortisol)^2^ β = 186.78; *p* = 0.568; F_(2,17)_ = 0.53; *p* = 0.596). Finally, there was no significant relationship between Δ Cortisol and deviation from optimal giving-up time in the first block of the task (prior to the stress manipulation; linear: F_(1,34)_ = 2.38; *p* = 0.132; quadratic: F_(2,33)_ = 2.30; *p* = 0.116). This demonstrates that the effect was a consequence of stress and not a trait variable that might correlate with the magnitude of the stress response.

We found a similar inverted-U shaped relationship between physiological responses to stress and our secondary measure of performance in the task: earnings post-manipulation. There was no significant linear relationship between Δ Cortisol and earnings (β = 1.18; *p* = 0.204; R^2^ = 0.0469; AIC = 101.255), but there was a significant quadratic relationship (Δ Cortisol β = 9.84; *p* = 0.002; 95% CI {3.83, 15.86}; (Δ Cortisol)^2^ β = −14.53; *p* = 0.005; 95% CI {-24.23, −4.83}; F_(2,33)_ = 5.69; *p* = 0.008; R^2^ = 0.2563; AIC = 94.327; likelihood ratio test: *χ*^2^ = 8.93; *p* = 0.003; Fig. 6B).^3^ When cortisol increased slightly, people earned more money in the task (regardless of task condition) but when it increased to higher levels, people earned less. Once again, this relationship did not hold in the control groups (Δ Cortisol β = −1.07; *p* = 0.255; (Δ Cortisol)^2^ β = −0.95; *p* = 0.768; F_(2,56)_ = 0.68; *p* = 0.5126; R^2^ = 0.0236; AIC = 187.85), or in non-responders within the stress group (Δ Cortisol < 0; N = 20; Δ Cortisol β = −19.12; *p* = 0.329; (Δ Cortisol)^2^ β = −93.00; *p* = 0.238; F_(2,17)_ = 0.99; *p* = 0.3923; R^2^ = 0.1042; AIC = 69.92).

### 3.5 Self-report questionnaire results

There were no significant correlations between giving-up time (AUC) or calibration of persistence (deviation from optimal) and scores on any of the self-report measures. There were, however, significant correlations among self-report measures (see Table 2 for correlation matrix).

**Table 2:** Correlations among self-report and behavioral measures.

*AUC*	*Deviation from optimal*	STAI-T	PSS	BDI-II	IUS	DoG	BIS-11	
	r = 0.02 p = 0.828	r = −0.03 p = 0.771	r = 0.05 p = 0.621	r = 0.02 p = 0.832	r = 0.05 p = 0.622	r = 0.11 p = 0.260	r = −0.06 p = 0.517	*AUC*
		r = −0.03 p = 0.756	r = −0.02 p = 0.867	r = −0.11 p = 0.246	r = −0.06 p = 0.544	r = 0.08 p = 0.415	r = −0.18 p = 0.054	*Deviation from optimal*
			r = 0.64^∗∗^ p < 0.001	r = 0.73^∗∗^ p < 0.001	r = 0.66^∗∗^ p < 0.001	r = −0.33^∗∗^ p < 0.001	r = 0.50^∗∗^ p < 0.001	STAI-T
				r = 0.64^∗∗^ p < 0.001	r = 0.58^∗∗^ p < 0.001	r = −0.28^∗^ p = 0.002	r = 0.44^∗∗^ p < 0.001	PSS
					r = 0.61^∗∗^ p < 0.001	r = −0.31^∗^ p = 0.001	r = 0.58^∗∗^ p < 0.001	BDI-II
						r = −0.11 p = 0.25	r = 0.28^∗^ p = 0.002	IUS
							r = −0.66^∗∗^ p < 0.001	DoG
								BIS-11

Pearson correlations among self-report and behavioral measures. Average giving-up time (AUC) and calibration of persistence (deviation from optimal giving-up time) post-manipulation were unrelated to scores on self-report questionnaire measures. STAI-T = State and Trait Anxiety Inventory – Trait version; PSS = Perceived Stress Scale; BDI-II = Beck Depression Inventory; IUS = Intolerance of Uncertainty Scale; DoG = Deferment of Gratification scale; BIS-11: Barratt Impulsiveness Scale (11-item). There were, however, significant correlations among self-report measures (*p < 0.01; **p < 0.001; p values are uncorrected for multiple comparisons; N = 115).

Given the finding that only thirty-six individuals within the stress group showed a cortisol response that was greater than zero, we investigated whether cortisol “responder” status was related to self-report measures of stress (unpleasantness rating and change in negative and positive affect). Within the stress group, cold pressor test unpleasantness ratings did not differ between responders and non-responders (t_57_ = 1.56; p = 0.124). The difference in negative affect from before to after stress also did not differ between these two subgroups (t_57_ = −0.71; p = 0.484). Finally, positive affect change also did not differ between these subgroups (t_57_ = −0.005; p = 0.996). Thus, it is not necessarily the case that people who reported feeling less stressed were the individuals who did not show an increase in cortisol following stress.

## 4 Discussion

The current study tested the effects of acute physical stress on a process that is emblematic of self-control: waiting for delayed rewards. We found that both persistence for delayed rewards and the calibration of persistence in response to environmental statistics were unaffected by acute stress. In an exploratory analysis of individual differences, we found that, among participants who responded physiologically to the stressor, a small increase in cortisol predicted better calibration of persistence, and a larger increase in cortisol was associated with worse calibration. In other words, there was an “inverted-U” relationship between performance in this task and cortisol response to stress. Given the post-hoc nature of this analysis, this result should be interpreted with caution. Nevertheless, it suggests an interesting hypothesis for testing in future studies.

Stress has been shown to impair performance on tasks that require cognitive control and flexibility (Arnsten, 2009, Otto et al., 2013, Plessow et al., 2012, Plessow et al., 2011, Raio et al., 2013), whereas it can actually improve performance on simpler, more well-rehearsed tasks (de Kloet et al., 1999, Diamond et al., 2007, Hartley and Adams, 1974, Sandi and Pinelo-Nava, 2007). This has been explained partly through neurobiological mechanisms. Cognitive control tasks rely on the prefrontal cortex, a region that is impaired following high levels of acute stress (Arnsten, 2009, Holmes and Wellman, 2009). One dominant theory of self-control has suggested that the role of regions involved in executive control (such as the PFC) is to facilitate persistence in the face of temptation (Heatherton and Wagner, 2011, Metcalfe and Mischel, 1999). Here we found that, regardless of the delay-timing statistics in the task, acute stress had no effect on persistence *per se*. This was the case both when looking at effects of stress overall, and when examining the relationship between average giving-up time and cortisol response. This null result is consistent with emerging literature suggesting that PFC control processes optimally integrate costs and benefits, rather than merely increase wait times (Berkman et al., 2017, Kurzban et al., 2013, McGuire and Kable, 2013, Shenhav et al., 2013). Previous evidence of the involvement of PFC in persistence might be better explained by its role in cost-benefit decision-making.

Most studies examining the effects of stress on self-control to date have utilized intertemporal choice tasks, which involve discrete choices between outcomes available at different points in time (e.g., “$10 today or $20 in 30 days”). There is some experimental evidence that stress leads to an increased likelihood to choose immediate rewards in these paradigms (Kimura et al., 2013), but the effect size is small (Fields et al., 2014), and the evidence is inconsistent. One study showed that there was no effect of laboratory-induced stress on intertemporal choices (Haushofer et al., 2013), and another showed that these decisions depended on individual differences in perceived stress (Lempert et al., 2012). The paradigm in the current study involved delays on the order of seconds, rather than days, and thus might involve a different decision process (Hayden, 2015, Jimura et al., 2013). Nevertheless, our null result regarding stress and persistence adds to the evidence that stress does not necessarily lead to more impulsive choice.

Self-control, though, can involve not just persistence, but the proper integration of contextual factors into the decision to persist. Giving up waiting for a reward can be conceptualized as a rational decision in response to certain environmental conditions. Here we found, consistent with previous studies (Fung et al., 2017, Massar and Chee, 2015, McGuire and Kable, 2015, McGuire and Kable, 2012), that individuals are capable of shifting their willingness to wait for rewards in response to the reward timing distributions they encounter. This capacity to calibrate persistence was preserved under acute stress, but the relationship between the cortisol response to stress and optimal calibration was curvilinear. If individuals showed a small physiological response to stress, then their performance improved, but at high levels of stress, their ability to calibrate persistence diminished. These results are in line with research showing that, in small amounts, stress facilitates performance on PFC-dependent tasks, but that more extreme stress leads to decrements in performance (Luksys and Sandi, 2011).

One limitation of the current study is that, whereas our stressor – the cold pressor test –induced significant increases in physiological and self-report measures of stress on average, many participants did not show a hormonal response. While variability in physiological responses allowed us to investigate individual differences, the relatively large number of non-responders may have limited our power to detect a relationship between cortisol response and behavior. Another limitation is that the only physiological measure of stress that we assayed was cortisol. Acute stress elicits both a fast sympathetic nervous system response and a slower HPA-axis response (Joëls and Baram, 2009). Here we were primarily interested in the HPA-axis response, but future studies will be needed to assess how stress might influence this decision process at different time points, and how different aspects of the stress response relate to these decisions. Finally, whether an individual perceives a stressful situation as a “challenge” or a “threat” (Blascovich et al., 1999), or as controllable or uncontrollable (Koolhaas et al., 2011), has been shown to impact later decisions (Kassam et al., 2009) and performance on cognitive tasks (Henderson et al., 2012, Jamieson et al., 2012, Jamieson et al., 2010). Here we did not probe for individual appraisals of the stressor, and we only examined one type of stress (physical pain), somewhat limiting the generalizability of our findings.

Self-control has often been defined as the ability to persist for future rewards, but persistence is not always optimal. Perhaps a more precise conceptualization of self-control is the ability to integrate information about the environment in order to flexibly adapt behavior. In line with this, we have shown that acute stress, which can impair controlled processes, has no effect on persistence, but does impair the calibration of persistence in individuals with a high cortisol response to stress. This work adds to our understanding of the mechanisms behind decision-making in complex environments, and how emotional states might affect these decisions.

## Footnotes

2 In individual differences analyses, participants with an AUC post-manipulation of <2.22 s were excluded (see *Behavioral Measures* in Method). Four of these participants were in the stress group, and one was in the control group.

3 When excluding four additional participants who were taking oral contraceptives (leaving N = 32), results remained similar for deviation from optimal giving-up time (linear: Δ Cortisol β = −9.15; *p* = 0.052; quadratic: Δ Cortisol β = −55.94; *p* = 0.001; (Δ Cortisol)^2^ β = 77.1; *p* = 0.003; F_(2,29)_ = 8.11; *p* = 0.0016; R^2^ = 0.3587; AIC = 182.51) and for earnings (linear: Δ Cortisol β = 1.32; *p* = 0.178; quadratic: Δ Cortisol β = 10.18; *p* = 0.004; (Δ Cortisol)^2^ β = −14.6; *p* = 0.008; F_(2,29)_ = 5.26; *p* = 0.0113; R^2^ = 0.2661; AIC = 85.02).
